# Sevoflurane and isoflurane inhibit KCl-induced, Rho kinase-mediated, and PI3K-participated vasoconstriction in aged diabetic rat aortas

**DOI:** 10.1186/s12871-021-01425-3

**Published:** 2021-09-01

**Authors:** Shaozhong Yang, Yu Liu, Shanshan Huang, Feihong Jin, Feng Qi

**Affiliations:** Department of Anesthesiology, Qilu Hospital, Cheeloo College of Medicine, Shandong University, No. 107, Wenhua West Road, Jinan, 250012 Shandong China

**Keywords:** Sevoflurane, Isoflurane, T2DM, PI3K, Rho kinase, KCl

## Abstract

**Background:**

The mechanism of volatile anesthetics on vascular smooth muscle (VSM) contraction in the setting of diabetes mellitus (DM) remains unclear. The current study was designed to determine the effects of sevoflurane (SEVO) and isoflurane (ISO) on phosphoinositide 3-kinase (PI3K) and Rho kinase (ROCK) mediated KCl-induced vasoconstriction in aged type 2 diabetic rats.

**Methods:**

KCl-induced (60 mM) contractions were examined in endothelium-denuded aortic rings from aged T2DM Otsuka Long-Evans Tokushima Fatty (OLETF) rats (65–70 weeks old), control age-matched nondiabetic Long-Evans Tokushima Otsuka (LETO) rats and young Wistar rats (6–8 weeks old). The effects of SEVO or ISO (1–3 minimum alveolar concentration, MAC) on KCl-induced vasoconstriction, as well as those of LY294002 (PI3K inhibitor) and Y27632 (ROCK inhibitor) were measured in aortic rings from the three groups using an isometric force transducer.

**Results:**

KCl induced rapid and continuous contraction of aortic smooth muscle in the three groups, and the contraction was more obvious in OLETF rats.

SEVO and ISO inhibited KCl-induced vasoconstriction in a concentration-dependent manner and were suppressed by LY294002 (10 µM) and Y27632 (1 µM). SEVO had a stronger inhibitory effect on the aortas of young Wistar rats than ISO, especially at 2 MAC and 3 MAC (*P* < 0.05). In aged rats, the inhibitory effect of ISO was stronger than that of SEVO, especially OLETF rats. There was no significant difference in the effects of different concentrations of ISO on arterial contraction among the three groups (*P* > 0.05). The effects of 1 MAC SEVO on Wistar rats and 3 MAC SEVO on OLETF rats, however, were noticeably and significantly different (*P* < 0.05).

Compared with the control condition, LY294002 and Y27632 had the most noticeable effect on the KCl-induced contraction of aortic rings in OLETF rats (*P* < 0.01).

**Conclusion:**

SEVO (3 MAC), ISO (1, 2, 3 MAC), LY294002 and Y27632 have more significant inhibitory effect on the contraction of vascular smooth muscle in aged T2MD rats. The mechanism of SEVO and ISO in vascular tension in T2DM is partly due to changes in PI3K and/or Rho kinase activity.

## Introduction

Diabetes mellitus (DM) is a chronic metabolic disease characterized by sustained hyperglycemia. It can be accompanied by a variety of systemic complications, such as cardiovascular disease, obesity and renal failure [1]. There are three types of diabetes: type 1 diabetes mellitus (T1DM), type 2 diabetes mellitus (T2DM) and gestational diabetes mellitus (GDM). Among all cases, 5–10% of patients have T1DM and are unable to produce insulin. T2DM, which is characterized by insulin resistance, accounts for approximately 90% of cases, and GDM accounts for less than 1% [2, 3]. In recent decades, the global incidence of all cases of diabetes has increased sharply, mainly due to the continuous increase in the incidence of T2DM [4]. During the perioperative period, diabetic patients are more likely to experience cardiovascular events such as myocardial ischemia [5]. These perioperative cardiac events may be related to abnormal myocardial blood flow caused by vascular dysfunction. Hyperglycemia is the main factor involved in vascular dysfunction in T2DM [6, 7].

Phosphoinositide 3 kinases (PI3Ks) have been indicated to be the key molecules of glucose homeostasis, and their dysfunction is related to an increase in blood glucose levels [8–10]. The importance of PI3Ks in patients with DM, however, is not limited to the regulation of glucose metabolism. It is also closely related to organ damage such as to the blood vessels, heart and brain [11–13]. Multiple studies have shown that DM can lead to a decrease in PI3K activity in the central nervous system; the upregulation of PI3K signaling in the brain can reverse the pathological consequences of DM, while the use of PI3K inhibitors can restore DM-related damage [14, 15].

In vivo, factors such as angiotensin II (AT-II), thrombin, and high levels of glucose in endothelial cells or smooth muscle cells can trigger the activation of Rho kinase [16]. Recent research shows that the Rho-associated kinase (ROCK) activity of circulating leukocytes in patients with T2DM is significantly increased, and inhibition of ROCK activity may play an additional role in the prevention and treatment of T2DM [17].

The key step in smooth muscle cell contraction is the phosphorylation of myosin light chain (MLC), which is mediated by Ca^2+^-calmodulin-activated MLC kinase. G protein-coupled receptor (GPCR) agonists interfere with this mechanism by regulating the intracellular Ca^2+^ concentration and ROCK activity. ROCK increases MLC phosphorylation (MLCP) by blocking the myosin-binding subunit of the enzyme, resulting in a negative effect on myosin phosphatase. ROCK can also promote the contraction of vascular smooth muscle (VSM) by enhancing the binding of VSM cell (VSMC) agonists to receptors [18].

SEVO and ISO are volatile anesthetics that inhibit the Ca^2+^ sensitization mechanism of animal arterial VSMCs [19]. The ROCK mechanism is the main intracellular pathway. A study showed that SEVO inhibited GTPγ S-stimulated Rho/ROCK-mediated vasoconstriction in isolated rat aortic VSM [20]. SEVO also inhibited Ang II-induced vasoconstriction by reducing protein kinase C (PKC) phosphorylation without affecting [Ca^2+^]i in VSM [21]. Authors of this study previously demonstrated that SEVO inhibited MLC and that PKC potentiated the phosphorylation of the inhibitory protein CPI-17 and myosin phosphatase target subunit MYPT1/Thr853 in response to Ang II. ISO also inhibited MLC phosphorylation in response to Ang II, which was associated with a decrease in MYPT1/Thr853 but not CPI-17 phosphorylation [22]. Moreover, we demonstrated that SEVO and ISO can mediate rat aortic vasodilation through the KCl/PI3K-C2α/Rho kinase/MYPT1/MLC pathway [23]. In addition, recent studies have confirmed that SEVO can inhibit the activity of Rho kinase in vascular smooth muscle cells and enhance vasodilation under PI3K inhibition [24].

Studies have shown that SEVO at clinical concentrations can significantly inhibit the response of nondiabetic rats to norepinephrine (NE), but it has no effect on diabetic rats [25]. Clinical studies have also demonstrated that the foot skin temperature of nondiabetic patients is higher than that of diabetic patients induced by ISO or SEVO anesthesia, suggesting that thermoregulation controlled by the sympathetic nervous system is impaired in diabetic patients [26]. These findings could be due to a change in the responsiveness of diabetic vessels to volatile anesthetics. The difference in responsiveness to volatile anesthetics between diabetic and nondiabetic patients and its mechanism have not been determined, especially in elderly patients with severe vascular disease. The aim of this study was to compare the effects of SEVO and ISO on PI3K and ROCK mediated KCl-induced aortic vasoconstriction in aged T2DM rats compared with age-matched nondiabetic rats and young Wistar rats.

## Methods

### Animals and experimental design

Male diabetic Otsuka Long-Evans Tokushima Fatty (OLETF) rats and Long-Evans Tokushima Otsuka (LETO) rats were purchased from Otsuka Pharmaceutical Co., Ltd. (Japan). Male LETO rats and young Wistar rats were selected as controls.

The OLETF rat is a spontaneous animal model of T2DM. It has the characteristics of congenital polyphagia, mild obesity, delayed hyperglycemia, hyperinsulinemia, hypertriglyceridemia and hypercholesterolemia, which is similar to the pathophysiological process of human T2DM [27]. All animals were maintained in temperature-controlled rooms with a 12 h/12 h circadian rhythm and were granted free access to standard food and water until the OLETF and LETO rats were 65–70 weeks old and the Wistar rats were 6–8 weeks old. The protocol was approved by the Shandong University Animal Care and Use Committee and Qilu Hospital of Shandong University Medical Ethics Committee (No. KYLL-2013–51), and all studies were conducted in accordance with the ‘‘Guide for the Care and Use of Laboratory Animals’’ published by the US National Institutes of Health [28]. All efforts were made to minimize the number of animals used in the study and the pain of the animals. The study was carried out in compliance with the ARRIVE guidelines.

### Oral glucose tolerance test

Diabetes was confirmed in all OLETF rats before purchase. To verify the quality of the model, an oral glucose tolerance test (OGTT) was conducted again before the experiment. After fasting the rats for 8–14 h (h), tail vein blood was collected, and fasting blood glucose was measured by the Johnson & Johnson rapid blood glucose meter test paper method. The rats were then given glucose (2 g/kg) by gavage, and the post-loading blood glucose was measured at 30, 60, 90 and 120 min. The criteria for DM [27] were as follows: peak blood glucose > 16.8 mmol/L, 2 h postprandial blood glucose > 11.2 mmol/L, and hyperglycemia > 1 week. After the confirmation of diabetes mellitus in OLETF rats, the rats in each group were used in the follow-up experiment.

### Vascular smooth muscle tissue preparation

OLETF, LETO and Wistar rats were anesthetized by intraperitoneal injection of pentobarbital sodium (40 mg/kg) and euthanized by exsanguination from the common carotid artery. The descending thoracic aorta was dissected carefully to remove adherent fat and connecting tissue and cut transversely into 3–4 mm long arterial rings. The endothelium was removed by gentle rubbing of the internal surface with a stainless steel needle. The arterial ring was then vertically mounted between two hooks, and the upper hook was connected to the lever of the equidistant force sensor.

### Isometric force measurement of vascular reactivity

Consistent with our previous method [23], KCl (60 mM) was used as an agonist for VSM contraction of the rat aorta in the three groups. Endothelium-denuded aortic rings were equilibrated under a resting tension of 3 g in Krebs bicarbonate solution (KBS) (in mmol/L: NaCl, 118.2; KCl, 4.6; CaCl_2_, 2.5; KH_2_PO_4_, 1.2; MgSO_4_, 1.2; NaHCO_3_, 24.8; and dextrose 10) at 37 °C and gassed with a mixture of 95% (v/v) O2 and 5% (v/v) CO_2_ with a fresh gas flow of 2 L/min. After 60 min of equilibration, and with the bathing fluid replaced every 20 min, the aortic rings were incubated with KCl (60 mM) to assess their overall contractile responsiveness. Removal of the endothelium was confirmed with 3 × 10^–7^ M phenylephrine-precontracted vessels that showed a lack of relaxation in the presence of 10^–5^ M acetylcholine.

To examine the effect of SEVO and ISO on KCl-induced contraction, after culture in KCl (60 mM) for 5 min, six aortic rings from each individual rat (*n* = 6) were randomly exposed to 0, 1, 2, and 3 minimum alveolar concentration (MAC) of each anesthetic, 10 μM LY294002, or 1 μM Y27632 for 15 min. SEVO or ISO was delivered into the gas mixture with a fresh gas flow of 2 L/min via a calibrated agent-specific vaporizer (Penlon, Abingdon, UK) to aerate the KBS with equivalent human MAC. The concentration of the produced gas mixture was monitored and adjusted using an Atom 303 anesthetic monitor (Atom, Tokyo, Japan). The concentration of SEVO in KBS was measured by gas chromatography (Shimazu Seisakusho, Tokyo, Japan). KCl-induced arterial systolic tension was measured by the isometric force method. The isometric forces of SEVO, ISO, LY294002 and Y27632 on KCl-induced contraction of rat descending aortic smooth muscle was expressed as a percentage relative to that induced by KCl (60 mM).

### Materials and reagents

Glucose is a prescription drug in the pharmacy department of Qilu Hospital of Shandong University. SEVO was purchased from Hengrui Pharmaceutical Company Limited (Jiangsu, China). ISO was obtained from Abbott Pharmaceutical Company Limited (Shanghai, China). Rho kinase inhibitors LY294002 and Y27632 were purchased from Selleck Chemical Company Limited (Shanghai, China). Blood glucose was measured with a Johnson & Johnson rapid blood glucose meter and test paper.

### Statistical analysis

SPSS 23.0 and GraphPad Prism 8.2.1 software were used for data analysis. The sample size (n) refers to the number of rats from which the aortas were harvested. The mean ± standard deviation was used for count data. The Shapiro Wilk test was used to determine whether the data conformed to a normal distribution. A nonparametric test was used for data not conforming to a normal distribution. Univariate and two-way ANOVA were used for comparisons between groups, and the Brown-Forsythe and Welch tests were used for data with uneven variance. A *p*-value < 0.05 was considered significant.

## Results

### Changes in the glucose tolerance curve in three groups of rats

**Fig. 1:**
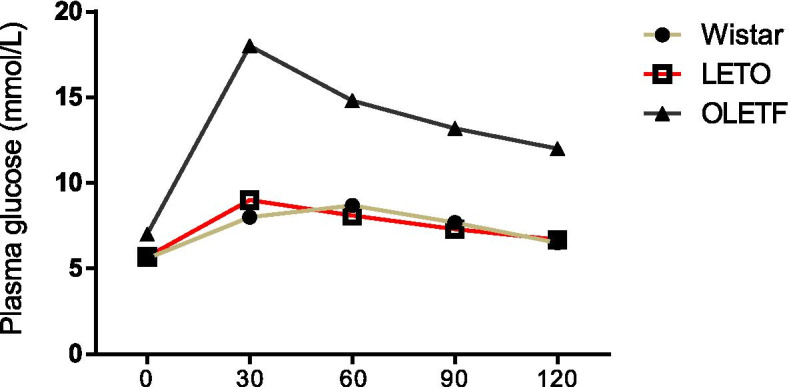
OGTT curve of young Wistar and aged OLETF and LETO rats (*n* = 12, each)

The results of the OGTT showed that the postprandial blood glucose of aged OLETF rats was significantly higher than that of the control groups (*P* < 0.01) and reached the highest value in 30 min. The peak blood glucose in LETO rats and Wistar rats was less than 10 mmol/L, but the peak time was different. The peak blood glucose in aged LETO rats was 30 min after a meal, and that in young Wistar rats was 60 min after a meal (Fig. 1).

### Effects of KCl (60 mM) on the contraction of aortic smooth muscle in three groups of rats

**Fig. 2:**
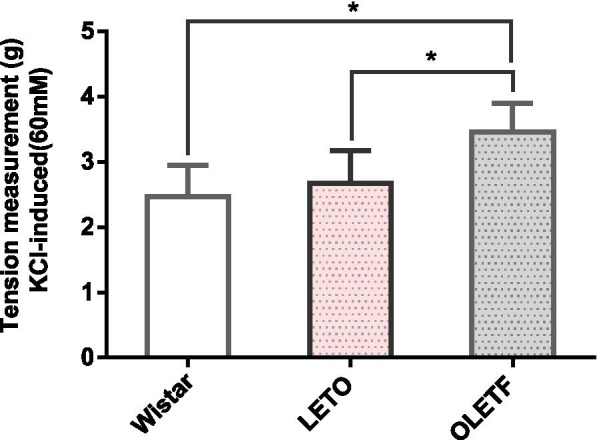
Effect of KCl (60 mM) on aortic smooth muscle contraction in aged OLETF and LETO rats and young Wistar rats (*n* = 12, each), *P < 0.05

KCl (60 mM) induced rapid and continuous contraction of aortic smooth muscle in the three groups. Compared with LETO and Wistar rats, the contraction of aortic smooth muscle in OLETF rats was more obvious (*P* < 0.01), but there was no significant difference between the control groups (Fig. 2).

### Effects of SEVO and ISO on KCl-induced contraction in rat aortic smooth muscle in the three groups

**Fig. 3:**
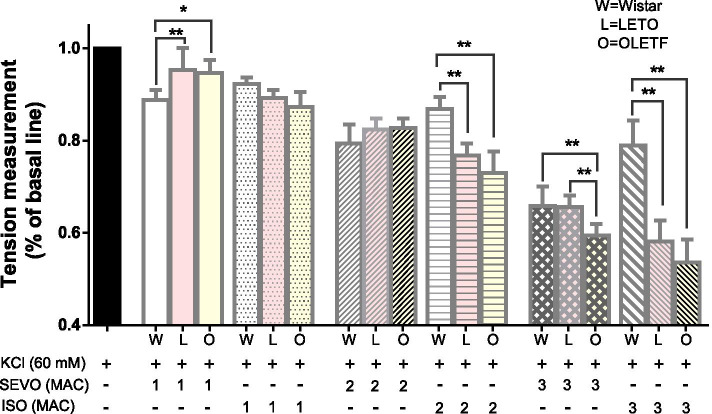
Effects of SEVO and ISO on KCl-induced contraction in OLETF and LETO rats and young Wistar rat aortic smooth muscle (*n* = 6, each). *P < 0.05, ** *P* < 0.01 versus the value in the presence of KCl without anesthetics

SEVO and ISO inhibited KCl-induced vasoconstriction in a concentration-dependent manner. SEVO had a more significant inhibitory effect on the aorta of young Wistar rats than ISO, with a significant difference between 2 and 3 MAC SEVO (*P* < 0.05). In the aged rat groups, the inhibitory effect of ISO was stronger than that of SEVO, and the OLETF group was more significantly affected than the LETO group; however, there was no significant difference between the two groups (Fig. 3).

1 MAC SEVO had the strongest inhibitory effect on aortic vasoconstriction in Wistar rats compared with OLETF and LETO rats (*P* < 0.05). In contrast, 2 MAC SEVO was not significantly different among the three groups. 3 MAC SEVO however had the strongest inhibitory effect on vasoconstriction in the OLETF group, which was significantly different from the LETO and Wistar groups (*P* < 0.05). There was no significant difference between the control groups. In contrast to SEVO, ISO had the weakest inhibitory effect on KCl-induced aortic vasoconstriction in young Wistar rats. Compared with the OLETF and LETO groups, the Wistar rats demonstrated a significant difference (*P* < 0.05), but no significant difference was observed between the OLETF and LETO groups (Fig. 3).

### Effect of LY294002 (10 μM) and Y27632 (1 μM) on KCl-induced contraction in rat aortic smooth muscle in the three groups

**Fig. 4:**
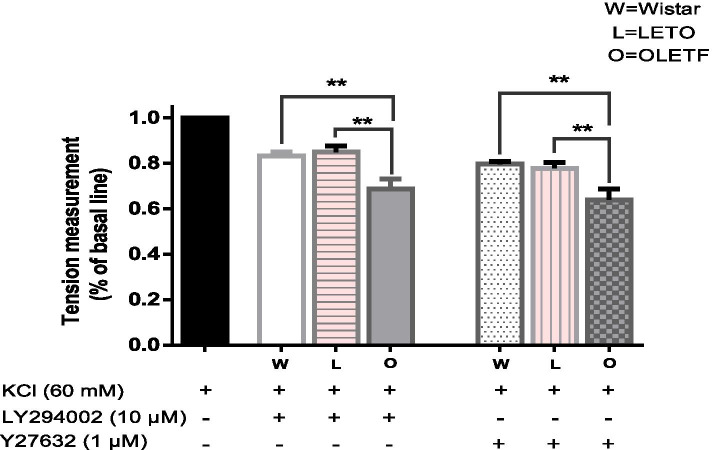
Effect of LY294002 (10 μM) and Y27632 (1 μM) on KCl-induced contraction in OLETF and LETO rats and young Wistar rat aortic smooth muscle (*n* = 6, each). *P < 0.05, **P < 0.01 versus the value in the presence of KCl without inhibitor

LY294002 (10 μM) and Y27632 (1 μM) inhibited KCl-induced aortic vasoconstriction in the three groups of rats. The inhibition degree was compared among the three groups. The OLETF group showed significantly higher inhibition than the LETO and Wistar groups (*P* < 0.01), but there was no significant difference between the control groups (Fig. 4).

## Discussion

The incidence rate of diabetes has increased suddenly in recent decades and is considered one of the major threats to human health in the twenty-first century [4]. It is estimated that 463 million people had diabetes in 2019, accounting for 9.3% of the global adult population; the prevalence of diabetes increased with age, resulting in a prevalence of 19.9% in 65- to 79-year-olds [29]. End-organ dysfunction is the main cause of mortality in diabetic patients, and vascular dysfunction is one of the major complications [2].

There are many main findings of this study. KCl significantly increased the rapid and continuous contraction of the aorta in aged T2DM rats. The clinical concentration of SEVO strongly inhibited KCl-induced vasoconstriction in young rats, while ISO had a stronger effect on vascular contraction in aged rats. LY294002 (10 μM) and Y27632 (1 μM) significantly increased the degree of aortic relaxation in OLETF rats.

Previous studies by the authors confirmed that PI3K is involved in the activation of Rho and the subsequent Rho kinase-dependent MLCP inhibition, resulting in vascular smooth muscle contraction [22]. The authors now demonstrated that SEVO and ISO attenuated KCl (60 mM)-induced arterial contraction in a concentration-dependent manner and that LY294002 (10 μM) and Y27632 (1 μM) also inhibit contraction. The KCl/PI3K-C2α/Rho kinase/MLCP/MLC pathway may mediate the intracellular mechanism of VSM contraction induced by volatile anesthetics [23].

Fujii et al. showed that SEVO did not alter the changes in Ca^2+^ in aortic cells of diabetic rats induced by NE [25], and SEVO at clinical concentrations could significantly inhibit the response of nondiabetic rats to NE but had no such effect on diabetic rats. Recent studies have shown that arterial reactivity to acetylcholine (ACh) and Ang II in diabetic mice is significantly changed, which is related to an increase in Rho kinase activity [30].

A clinical study revealed that, compared to the healthy control group, ROCK activity in the peripheral blood of patients with T2DM was significantly increased and correlated with the blood glucose level. The phosphorylation of upstream and downstream cascade proteins was also increased, suggesting that specific inhibition of ROCK activity may play an additional role in the prevention and treatment of T2DM [17]. Hofni A et al. demonstrated that the Rho/ROCK pathway plays a key role in the pathogenesis of diabetic angiopathy. By interfering with the Rho/ROCK pathway, selective ROCK inhibitors can reduce vascular dysfunction caused by diabetes [31].

Diabetes can reduce PI3K activity in the central nervous system; the upregulation of PI3K signaling in the brain can reverse the pathological consequences of diabetes, and the use of PI3K inhibitors can reduce diabetes-related damage [14, 15]. In the case of obesity and hyperinsulinemia, a decrease in PI3K activation may be a key step in supporting the increase in insulin-induced vasoconstriction. Several studies have confirmed that insulin regulates vasomotor tension through vasodilator and vasoconstrictor signaling pathways. In the case of sustained insulin signaling, impaired PI3K activation seems to be a necessary feature for inducing insulin-mediated vasoconstriction [32]. Recent studies have shown that hyperglycemia can inhibit the PI3K/Akt signaling pathway in the aorta and vascular smooth muscle cells in diabetic mice and activate the PI3K/Akt signaling pathway, which can play a protective role in diabetes-mediated aortic hypercontraction [33].

Age and diabetes cause changes in arterial reactivity to different volatile anesthetics. Age can lead to changes in the function of the central arteries rather than the peripheral arterioles [34]. Long-term hyperglycemia can change the mechanism of arterial smooth muscle contraction through continuous insulin signaling [35]. Combined with the findings of our previous studies, our results indicate that PI3K and Rho kinase activity changes in diabetic vessels.

This study has some limitations. PI3K can activate endothelial nitric oxide synthase (eNOS), leading to production of the vasodilator NO. Endothelial ROCK may act to suppress PI3K activity [36]. To avoid the effect of endothelium-derived vasodilators, we used endothelium-denuded aortic strips or rings for this study.The aorta is a elongated vessel, and the experimental data from these smaller transverse segments may not directly explain the effect in vivo. 1, 2 MAC SEVO has stronger inhibitory effect on young rats. Considering the relationship between different types of rats and poor elasticity of aging blood vessels. However, the specific mechanism needs to be further studied. SEVO however has a lower inhibitory effect on aged blood vessels than ISO, which is consistent with the pharmacology of volatile anesthetics [37]. The specific effect of volatile anesthetics on aged diabetic vasculature is related to changes in PI3K and Rho kinase activities. Nevertheless, we did not analyze the concentration and activity of PI3K and Rho kinase in blood vessels by Western blotting, which needs further study.

In conclusion, KCl significantly increased the rapid and continuous contraction of aortic rings in aged T2DM rats. The clinical concentration of ISO has a smaller inhibitory effect on blood vessels in young rats, while SEVO has a smaller inhibitory effect in aged rats. The mechanism of SEVO and ISO in vascular tension in T2DM is due to changes in PI3K and/or Rho kinase activity. The cellular mechanism of the vascular effect of volatile anesthetics on T2DM seems to be complex and remains to be elucidated.

## Data Availability

The datasets used in the current study are available from the corresponding author upon reasonable request.

## Abbreviations

SEVO: Sevoflurane
VSM: Vascular smooth muscle
ISO: Isoflurane
MAC: Minimum alveolar concentration
T2DM: Type 2 diabetes mellitus
PI3K: Phosphoinositide 3 kinase
Ang II: Angiotensin II
ROCK: Rho-associated kinase
MLC: Myosin light chain
GPCR: G protein-coupled receptors
MLCP: MLC phosphatase
MYPT1/Thr853: Myosin phosphatase target subunit/Thr853
PI3K-C2α: Class II phosphoinositide 3-kinase α-isoform
NE: Norepinephrine
OGTT: Oral glucose tolerance test
